# Extrachromosomal circular DNA and their roles in cancer progression

**DOI:** 10.1016/j.gendis.2023.101202

**Published:** 2023-12-22

**Authors:** Siqi Zheng, Yunong Li, Lin Wang, Qian Wei, Minjie Wei, Tao Yu, Lin Zhao

**Affiliations:** a Department of Pharmacology, School of Pharmacy, China Liaoning Key Laboratory of Molecular Targeted Anti-Tumor Drug Development and Evaluation, Liaoning Cancer Immune Peptide Drug Engineering Technology Research Center, Key Laboratory of Precision Diagnosis and Treatment of Gastrointestinal Tumors, Ministry of Education, China Medical University, Shenyang, Liaoning 110122, China; b Department of Medical Imaging, Cancer Hospital of China Medical University, Liaoning Cancer Hospital & Institute, Shenyang, Liaoning 110042, China

**Keywords:** Alternative splicing, Cancer stem cell, Extrachromosomal circle DNA, Protein–protein interaction, Tumor heterogeneity, Tumor immune microenvironment

## Abstract

Extrachromosomal circular DNA (eccDNA), a chromosome-independent circular DNA, has garnered significant attention due to its widespread distribution and intricate biogenesis in carcinoma. Existing research findings propose that multiple eccDNAs contribute to drug resistance in cancer treatments through complex and interrelated regulatory mechanisms. The unique structure and genetic properties of eccDNA increase tumor heterogeneity. This increased diversity is a result of eccDNA's ability to stimulate oncogene remodeling and participate in anomalous splicing processes through chimeric cyclization and the reintegration of loop DNA back into the linear genome. Such actions promote oncogene amplification and silencing. eccDNA orchestrates protein interactions and modulates protein degradation by acting as a regulatory messenger. Moreover, it plays a pivotal role in modeling the tumor microenvironment and intensifying the stemness characteristics of tumor cells. This review presented detailed information about the biogenesis, distinguishing features, and functions of eccDNA, emphasized the role and mechanisms of eccDNA during cancer treatment, and further proposed the great potential of eccDNA in inspiring novel strategies for precision cancer therapy and facilitating the discovery of prognostic biomarkers for cancer.

## Introduction

Cancer therapy continues to grapple with the significant hurdle of drug resistance, a complex issue with unclear mechanisms at play. Research indicates that extrachromosomal circular DNA (eccDNA) plays a pivotal role in the heterogeneity observed within tumors. This diversity facilitates the swift adaptability of malignant cells when confronted with fluctuating therapeutic and environmental challenges.^1^^,^^2^ In addition to frequently carrying oncogenes, eccDNA partakes in a myriad of cellular functions, including cell proliferation, apoptosis, modulation of the tumor-immune microenvironment, and sustaining tumor stem cells.^3^, ^4^, ^5^, ^6^ eccDNA plays a central role in irregular oncogene shedding and oncogene silencing processes, culminating in the development of treatment resistance and tumor recurrence. Also, by mediating chromatin via RNA polymerase II-driven interactions, eccDNA contacts specific chromosomal genes, functioning as mobile enhancers to up-regulate the expression of tumorigenic pathway genes.^7^ Moreover, eccDNA, especially when size-limited, tends to harbor fewer full-length protein-coding sequences. Instead, they frequently encode regulatory short RNAs, such as microRNAs and siRNAs, which play a vital role in the regulation of gene expression and tumorigenesis.^8^ Notably, these small circular DNA structures could also act as “sponges” for transcription factors, exerting an indirect influence on gene expression.^9^

This review will focus on the two main categories of research on eccDNA and its effects on tumors: functional and mechanistic studies. The manuscript will commence by delving into the present knowledge concerning ec-cDNA's influence on distinct cancer types. The spotlight will focus on its significant contribution to tumor heterogeneity, preservation of tumor stem cells, and modulation of the tumor microenvironment. Subsequent sections will explore the intricate manner in which eccDNA regulates both proto-oncogenes and tumor suppressor genes. Lastly, the discussion will pivot to the effects of eccDNA on protein–protein interactions, underscoring its association with recognized oncogenic pathways.

## Biogenesis and structure of eccDNA

### Biogenesis

eccDNA formation typically stems from chromosomal DNA damage and aberrations in various DNA repair pathways. Four primary pathways are implicated in this process: double-strand breaks, chromothripsis, breakage-fusion-bridge, and the exosome pathway.^9^, ^10^, ^11^, ^12^, ^13^, ^14^ Classification of eccDNA has led to the identification of five types: small polydisperse DNA, microDNA, telomeric loops, extrachromosomal rDNA loops, and ECDNA.^15^^,^^16^ Due to the absence of a standardized classification system for eccDNA, we will refer to all types collectively as eccDNA in this review.

### Structure

eccDNA has its origins scattered across myriad unique genomic locations and is predominantly concentrated within particular regions. Notably, these regions comprise untranslated areas and those with a high GC content. A compelling observation is the positive correlation between eccDNA's base content and the ratio of the genes it encodes.^16^ Predominantly, the sequences constituting eccDNA arise from helices, exons, as well as cut-and-paste transposons.^17^ The dimensional variation of eccDNA is noteworthy, with its size oscillating between a few bases to several hundred thousand bases. It is evident that the majority of eccDNA measures below 1000 bp, with a staggering 99% not exceeding 25 kb in length.^16^^,^^18^ In the realm of normal cells, the prevailing eccDNA dimensions are concise, generally not surpassing 500 bp.^18^ eccDNA in tumor cells is characterized by preferred end coordinates and somatic variants and is smaller in size compared with eccDNA in normal cells.^19^^,^^20^ In conclusion, the structure and size of eccDNA are tissue- and disease-specific. These findings provide new ideas for characterizing the origin of eccDNA, exploring its functions, and facilitating cancer therapy.

### eccDNA in cancer

Research conducted in Mischel's laboratory unveiled that resistance to epidermal growth factor receptor (EGFR) inhibitors in glioblastoma could be attributed to the reversible loss of *EGFRvIII* gain-of-function mutations on eccDNA.^21^ Additionally, eccDNA can cause a considerable surge in the copy number of oncogenes in tumor cells, a feat challenging for chromosomal amplification to achieve.^2^ Interestingly, eccDNA does not carry mitotic elements, enabling it to realize targeted enrichment in tumor cells via asymmetric distribution.^22^ eccDNA has a host of functions, such as communication,^23^ aging,^24^ and acting as a molecular sponge,^8^ among others. Within tumor tissues, eccDNA enhances the heterogeneity of tumor cells and engages in various malignant evolutionary processes, such as proliferation, apoptosis, tumor stem cell preservation, and tumor immune microenvironment.^3^, ^4^, ^5^, ^6^ Next, we will systematically discuss the function of eccDNA during tumor progression and demonstrate the potential of eccDNA as a tumor biomarker and therapeutic target.

### eccDNA associates tumor heterogeneity

Tumors are heterogeneous cell populations that drive tumor progression and influence tumor outcomes.^25^ As a result of factors such as clonal variation within cancer cells, intra-tumor heterogeneity is further enhanced.^26^ Adapting and selecting the most suitable tumor clones can be facilitated by random mutations in individual tumor cells. It is predicted that clones that gain a growth advantage will gradually expand, while less adaptive ones will be eliminated in a competitive manner and eventually go extinct.^27^ The lack of centromere and unequal divisions of eccDNA may be the cause of the tumor heterogeneity that rises before and during tumorigenesis. Cells are believed to adapt better if they contain a large number of copies of these extrachromosomal elements.^10^ The distribution of eccDNA in progeny cells more closely resembles a binomial random or Gaussian distribution.^2^ eccDNA always reversibly drives the up-regulation or inhibition of mutant *EGFR*, thereby leading to a growth advantage in tumors with cells resistant to EGFR-targeted tyrosine kinase inhibitors after treatment, in glioblastoma cells.^21^ Thus, the asymmetric transgenerational nature of eccDNA enhances tumor heterogeneity and drives malignant progression.

### eccDNA regulates tumor cell stemness

**Figure 1:**
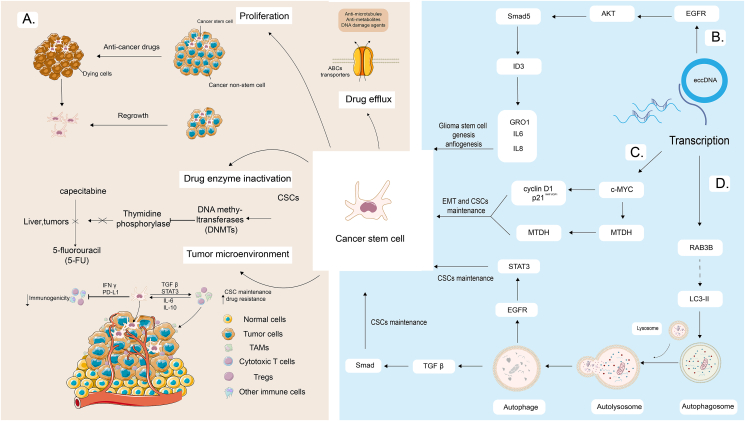
eccDNA-mediated increase in tumor cell stemness in tumor cells. **(A)** Overview of mechanisms by which tumor stem cells accelerate tumor progression. **(B)** eccDNA-mediated *EGFR* expression on tumor cells initiates glioma stem cell genes and enhances glioma stem cell stemness. **(C)** eccDNA-induced *c-myc* amplification regulates the cell cycle progression of glioma cancer stem cells and activates the MTDH-Twist1 signaling pathway, enhancing tumor cell stemness. **(D)** eccDNA-mediated transcription of the tumor cell autophagy gene *RAB3B* enhances tumor cell stemness through the EGFR/Stat3 and Tgfβ/Smad signaling pathways.

Cancer stem cells (CSCs), known for their self-renewal ability and potential to differentiate into a variety of cell types, contribute significantly to tumor heterogeneity.^28^ Mutations that affect adult stem cells, which ordinarily oversee organogenesis and tissue homeostasis, can give rise to CSCs.^29^, ^30^, ^31^ In addition to their inherently slow proliferation rates, CSCs have developed several cellular processes to withstand therapeutic intervention, including drug efflux, and inactivation of drug enzymes, among other mechanisms (Fig. 1A).^32^, ^33^, ^34^, ^35^ Furthermore, the microenvironment wherein CSCs evolve profoundly impacts cancer initiation, progression, and chemoresistance.^36^ Within the CSC's microenvironment, cancer-associated fibroblasts, mesenchymal stem cells, endothelial cells, and immune cells collectively foster cancer progression and enhance chemotherapy resistance.^37^ Interestingly, it has been found that eccDNA amplification in genes like *EGFR*, *c-MYC*, and *RAB3B*, which are associated with autophagy, can intensify the "stemness" of tumor cells. This can aid the maintenance of malignancy and expedite the development of tumor cells. Thus, the role of eccDNA in enhancing stemness underscores its significance in the complex puzzle of cancer biology. Further studies on the interplay between CSCs, their microenvironment, and eccDNA could illuminate new avenues for therapeutic interventions.

### EGFR signaling pathway regulated by eccDNA in CSCs

EGFR plays a crucial role in promoting the expression of inhibitory differentiation factor 3. This in turn leads to the initiation of cytokines such as interleukin (IL)-6 and IL-8. These cytokines are instrumental in up-regulating genes in glioma stem cells (GSCs) which are known for regulating proliferation, angiogenesis, and the acquisition of GSC characteristics (Fig. 1B).^38^ eccDNA is known to serve as an amplification platform for the oncogene *EGFR* (including its endogenous enhancer) and rapidly amplify the *EGFR* oncogene and even surpass the copy number of *EGFR* in chromosomal amplification, leading to aberrant *EGFR* expression.^2^ Similarly, eccDNA not only encodes the tumor-specific and highly transcribed oncogene *EGFR* but also amplifies oncogenes at a higher copy number compared with those amplified by linear chromosomes.^4^ Additionally, activating mutations of *EGFR* are predominantly found on eccDNA structures, reinforcing its role in oncogenic amplification. Therefore, eccDNA significantly contributes to the promotion of GSC self-renewal, augmentation of tumor cell stemness, and influencing therapeutic efficacy through *EGFR* up-regulation. A better understanding of these mechanisms provides valuable insights for future therapies against cancer.

### *C-myc*-related signaling regulated by eccDNA in CSCs

In the realm of oncology, eccDNA plays an instrumental role in augmenting proteins of the Myc family, notably C-myc, N-myc, and L-myc. These proteins stand at the forefront of processes leading to tumor emergence and subsequent progression. Notably, C-myc frequently emerges as the prime driver in human tumorigenic processes. An intriguing observation is the selective capture of c-myc-enriched eccDNA through specific nuclear protrusions during the S-phase. This process culminates in the micronucleation, positioning, and replication of c-myc eccDNA specifically in the nuclear margins of certain colon cancer cell models.^39^ Parallelly, eccDNA has the capability of amplifying *c-myc*, and the amplified copies of *c-myc* on eccDNA could be eliminated after administration of the anti-cancer drug hydroxyurea, thereby decreasing tumorigenicity both *in vitro* and *in vivo*.^40^ The amplification of c-myc stands out as instrumental in the sustenance of GSCs. This is attributed to its influence on the progression dynamics of the GSC cell cycle, chiefly by modulating the expressions of both cyclin D1 and p21^WAF1/CIP1^.^41^ Furthermore, in triple-negative breast cancer, *c-myc* transcription influences MTDH expression, leading to the stimulation of Twist1 signaling. This process is believed to maintain the tumor's epithelial–mesenchymal transition characteristics and CSC-like status, thereby bolstering tumor cell stemness and the malignant progression of the tumor (Fig. 1C).^42^

### Autophagy-related gene RAB3B signaling regulated by eccDNA in CSCs

eccDNA plays a crucial role in the resistance to cisplatin exhibited by hypopharyngeal squamous cancer, in which there is a correlation between the eccDNA-encoded gene RAB3B and the promotion of cellular autophagy, which stimulates autophagy and promotes the conversion of LC3-I into LC3-II in hypopharyngeal squamous cancer cells, as well as down-regulates p62 in the drug-resistant cells, which results in the production of more autophagic vesicles and ultimately induces hypopharyngeal squamous cancer cells to become resistant to cisplatin^43^. Meanwhile, autophagy is recognized as a central process in supporting and intensifying the aggression of CSCs (Fig. 1D). Autophagy compromises the tumorigenicity of two different types of breast cancer stem cell-like cells, highlighting the role of EGFR/Stat3 and TGFβ/Smad signaling.^44^ In GSCs, autophagy also modulates migration and invasion, with an increase in autophagy regulators DRAM1 and SQSTM1 influencing the expression of mesenchymal factors.^45^ Moreover, autophagy can promote the transformation of non-CSCs into CSCs, potentially via the IL-6-JAK2-STAT3 signaling pathway.^46^ From this evidence, it can be inferred that eccDNA, through the amplification of *RAB3B*, can induce autophagy in tumors, which could consequently enhance tumor cell stemness, leading to drug resistance of cancer cells and affecting the efficacy of tumor therapy.

### eccDNA regulates the tumor immune microenvironment and influences therapeutic efficacy

The success of tumor immunotherapy is significantly influenced by the tumor microenvironment, specifically the tumor immune microenvironment. The tumor immune microenvironment is characterized by the milieu within which cancer cells reside, encompassing elements such as circulating blood vessels, immune cells, fibroblasts, inflammatory cells derived from bone marrow, signaling molecules, and components of the extracellular matrix.^47^ There is a constant reciprocal interaction between cancer cells and their surrounding microenvironment. Equally important is how the immune system within the microenvironment influences the evolution and propagation of cancer cells.^48^, ^49^, ^50^, ^51^ Immune cells stimulated by eccDNA have been found to secrete type I interferon, IL-6, and tumor necrosis factor-alpha (TNF-α), which in turn, influence the effectiveness of tumor immunotherapy. Notably, eccDNA has been linked to the amplification of the neuroblastoma-related gene, *SOX11*. This overexpression obstructs the maturation process of B cells within the tumor immune microenvironment, leading to a decrease in the effectiveness of immunotherapy. Moreover, neurotrophin-3 expression, regulated via eccDNA, has been found to limit the phagocytic capabilities of microglia and deactivate the immune response within the brain, consequently impacting the effectiveness of tumor immunotherapy.

### Type I interferons regulated by eccDNA in the tumor microenvironment and tumor immunity

**Figure 2:**
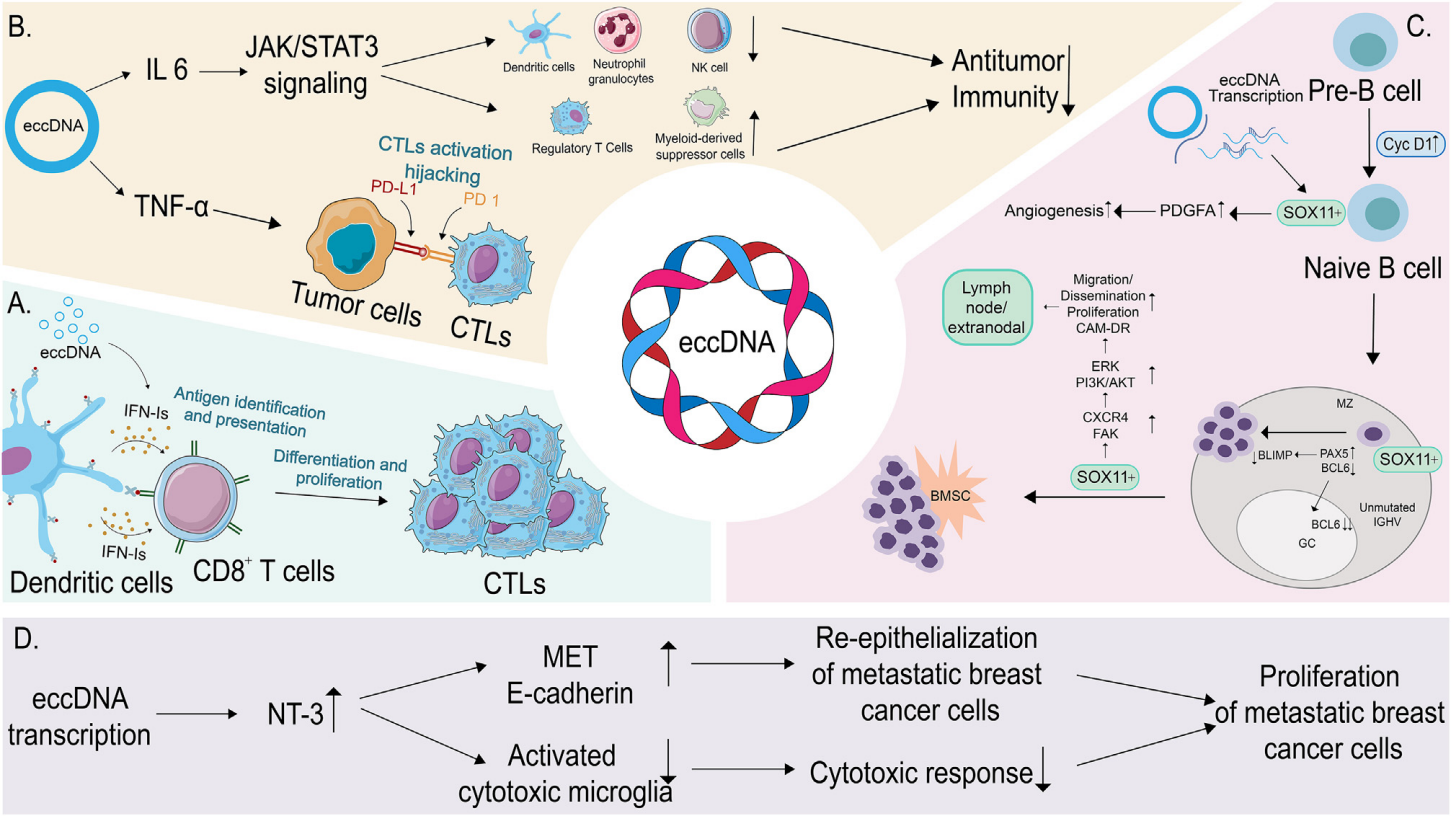
eccDNA regulates the tumor immune microenvironment (TIME) and influences therapeutic efficacy. **(A)** Cytokine type I interferons (IFN-Is) produced by eccDNA-stimulated immune cells indirectly enhance the cellular response of cytotoxic T cells (CTLs) by enhancing cross-presentation of tumor antigens by dendritic cells (DCs). **(B)** eccDNA induces up-regulation of interleukin (IL)-6 and tumor necrosis factor-alpha (TNF-α). IL-6 is involved in regulating tumor-infiltrating immune cell activity, leading to down-regulation of anti-tumor immunity. TNF-α mediates programmed death-ligand 1 (PD-L1) expression on tumor cells, which binds to programmed death 1 (PD-1) expressed CTLs, thereby inactivating them. **(C)** eccDNA-mediated SOX11 expression in tumor cells inhibits the development of B cells in the tumor immune microenvironment and enhances the immunosuppressive effects of the tumor immune microenvironment. **(D)** eccDNA-induced neurotrophin-3 (NT-3) up-regulation suppresses brain immune responses and promotes brain metastatic breast cancer cell growth.

Through the activation of the stimulator of interferon genes (STING)-dependent cytosolic DNA sensing pathway, eccDNA elicits immune responses (Fig. 2A).^52^ The STING pathway operates as an adaptor, signaling from the primary cytosolic detector of double-stranded DNA, the cyclic GMP-AMP synthase. This pathway phosphorylates TBK1, which subsequently activates the transcription factor interferon regulatory factor 3 leading to the expression of type I interferons, such as interferon β.^53^, ^54^, ^55^, ^56^ The cyclic GMP-AMP synthase, the cytosolic receptor for eccDNA, is responsible for inducing the type I interferon response in human fibroblasts.^52^ *In vitro* studies have demonstrated an amplification of the anti-tumor effector function of CD8^+^ T cells in cancer via type I interferons.^57^ Moreover, type I interferons directly influence the survival of intratumoral cytotoxic T cells. Indirectly, it bolsters the responses of cytotoxic T cells by improving the cross-presentation of tumor antigens by dendritic cells.^58^, ^59^, ^60^ Collectively, it can be concluded that the type I interferons produced by eccDNA-stimulated immune cells enhance the anti-tumor effects of cytotoxic T cells, thereby improving the efficacy of immunotherapy.

### IL-6 and TNF-α regulated by eccDNA in the tumor microenvironment and tumor immunity

eccDNA incites immune cells to generate IL-6 alongside TNF-α, both intensifying immunosuppressive responses and promoting tumorigenic actions (Fig. 2B).^61^ Elevated IL-6 levels lead to JAK/STAT3 pathway activation, which unfavorably affects diverse immune cells like neutrophils, natural killer cells, and dendritic cells, ultimately diminishing anti-tumor immune defenses.^62^, ^63^, ^64^, ^65^ Additionally, STAT3 plays a pivotal role in the augmentation of myeloid-derived suppressor cells and Tregs.^66^^,^^67^ Produced predominantly by macrophages, TNF-α acts to reinforce and maintain programmed death-ligand 1 protein expression and plays a role in modulating cytotoxic T cell activation, cumulatively resulting in cancer-associated immunosuppression.^68^ In essence, eccDNA-mediated actions can pave the way for a notably immunosuppressive tumor milieu, undermining tumor immunotherapy's efficacy.

### *SOX11* regulated by eccDNA in the tumor microenvironment and tumor immunity

Neuroblastoma-associated genes such as *SOX11* are amplified and cyclized by eccDNA, which is derived exclusively from the amplified alleles, whereas eccDNA contributes considerably to genome amplification, and its mediated extrachromosomal cyclization is a potential driver of high levels of focal genome amplification, which promotes high expression of *SOX11* (Fig. 2C).^3^ Moreover, up-regulation of *SOX11* affects B-cell development by preserving PAX5 expression and inactivating Blimp1, thereby preventing B cells from undergoing terminal differentiation, whereas antitumor genes are down-regulated in SOX11-expressing mantle cell lymphoma with significant immune infiltration.^69^, ^70^, ^71^ Consequently, eccDNA-mediated *SOX11* upregulation enhances the tumor immune microenvironment's immunosuppressive effects and reduces tumor immunotherapy efficacy.

### Neurotrophin-3 regulated by eccDNA in the tumor microenvironment and tumor immunity

Neurotrophic factor-3 (NT-3), an eminent neurotrophic factor in neuroblastoma, is integrated into circular DNA and subsequently amplified into eccDNA, with high expression levels in amplified eccDNA attributed to single alleles located in larger eccDNA.^3^ NT-3 plays a pivotal role in impeding microglia activation, and microglial cells pre-incubated with NT-3 displayed a reduction in the production of inducible nitric oxide synthase, nitric oxide, TNF-α, IL-1β, and a decline in microphagocytic capacity.^72^ Additionally, in the context of brain metastases from breast cancer, an increase in NT-3 secretion leads to a phenotypic transition of cells from mesenchymal-like to epithelial-like, resulting in an augmentation in the expression of human epidermal growth factor receptor 2 and E-calmodulin at cell–cell junctions.^73^ Consequently, the elevated expression of NT-3 mediated by eccDNA helps regulate the tumor microenvironment at brain metastasis sites. This, in turn, fosters the survival of metastatic tumor cells, thereby diminishing the effectiveness of tumor immunotherapy (Fig. 2D).

## The regulation mechanisms of gene expression by eccDNA in cancer cells

### Transcriptional regulation of eccDNA

#### eccDNA participates in abnormal splicing of proto-oncogene

**Figure 3:**
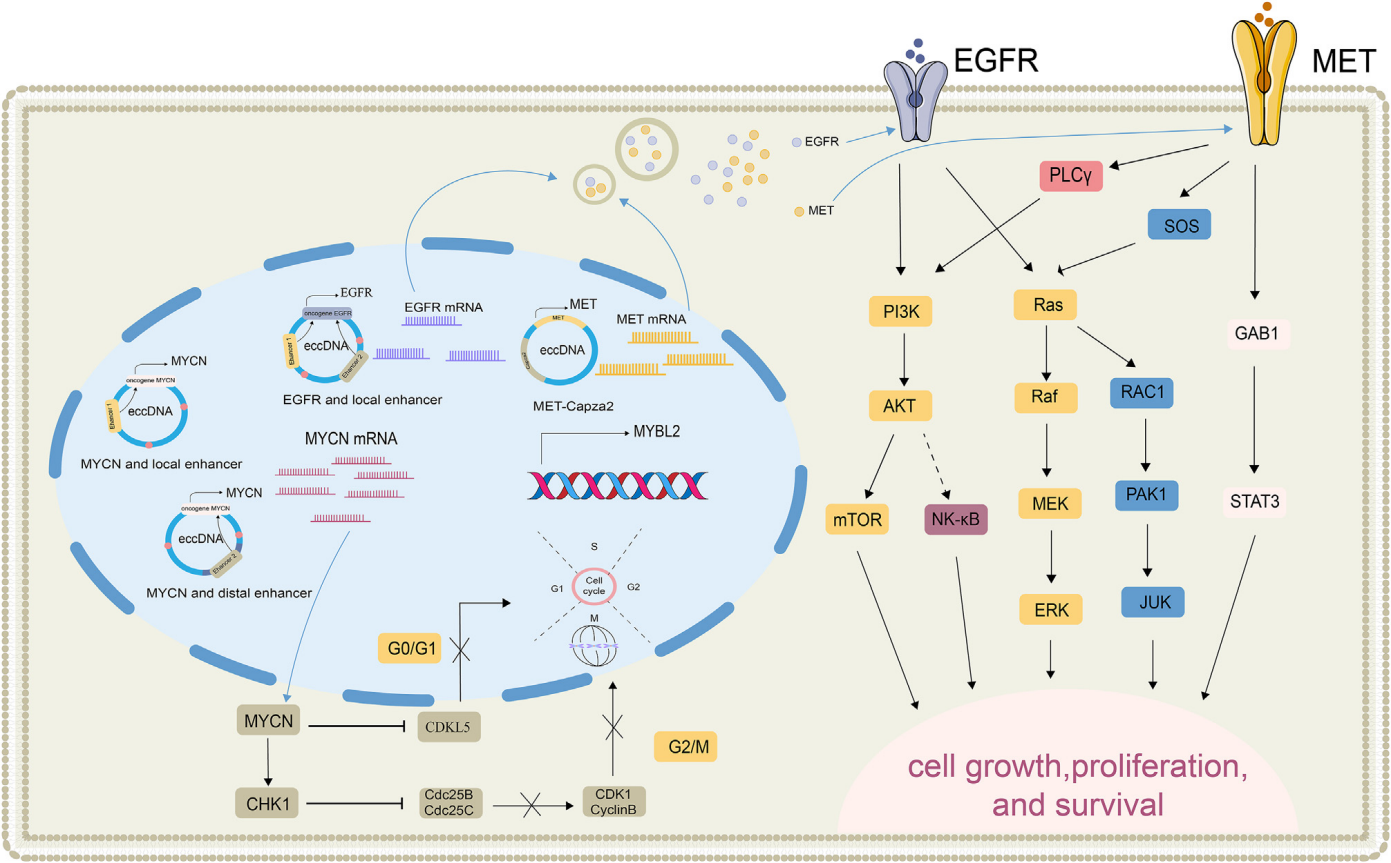
eccDNA mediates the production of aberrant oncogene shedders that activate survival signaling pathways mediating cancer progression. eccDNA aberrant shedder production directly leads to aberrant expression of multiple oncogenes (EGFR, MET, MYCN), activates PI3K/AKT, RAS/ERK/MAPK, Wnt/β-catenin, and other signaling pathways, and accelerates tumor progression.

eccDNA-mediated abnormal splicing includes the joining of oncogene fragments and enhancers from adjacent noncoding regions for cyclization, the joining of oncogene fragments and distant enhancers to make a larger extrachromosomal DNA loop, and the interaction of eccDNA with chromosomal DNA or other eccDNA as mobile enhancers. This variable shear heterogeneity mediated by eccDNA leads to oncogene overexpression, which also reflects its key role in oncogenic transcriptional regulation, provides a selective advantage for tumor cell survival, and mediates tumor cell drug resistance (Fig. 3). For example, the *EGFR*-enhancer, *MET*-*Capza2*, is formed by eccDNA-mediated formation. In addition, eccDNA can also reintegrate into the chromosomal genome, thus leading to the aberrant activation of oncogenes in its vicinity, such as *MYCN*.

#### Abnormal *EGFR*-enhancer splicing mediated by eccDNA

Localized aberrant enhancer-oncogene shear formation is involved in the amplification of *EGFR* in glioblastoma: *EGFR* localized on eccDNA co-amplifies with its upstream enhancer and that aberrant shedders of the reformed *EGFR*-enhancer promote cancer cell survival.^6^ Thus, the selective pressure results in the formation of aberrant shedders and promotes the co-amplification of oncogenes with regulatory elements that alter oncogene expression. EGFR serves as a receptor for epithelial growth factor, playing a pivotal role in cell proliferation and signaling.^74^ Upon overexpression, EGFR is activated and subsequently stimulates key intracellular signaling pathways. These include the RAS-RAF-MEK-ERK pathway (also known as the MAPK/ERK pathway), which is integral to cell survival, and the PI3K-AKT-mT0R pathway.^75^, ^76^, ^77^ Therefore, eccDNA-mediated irregular shedder production of the oncogene *EGFR* leads to its anomalous activation. This heightens the proliferative and survival capabilities of tumor cells, thereby fostering drug resistance within these cells.

#### Abnormal *MET*-*Capza2* splicing mediated by eccDNA

In glioblastoma, the *MET*-*Capza2* reassembly is mediated by eccDNA, and this fusion transcript prompts the overexpression of the *MET* oncogene. The eccDNA-mediated *CAPZA2*-*MET* fusion variants are homozygous transcripts, encompassing exon 1 of *CAPZA2* along with exons 3 and 6 of *MET*.^5^ MET, also recognized as c-Met or the HGF receptor, is a tyrosine kinase receptor.^78^^,^^79^ The variable splice heterogeneity instigated by eccDNA fosters *MET* overexpression. Consequently, the overexpressed MET proteins aberrantly activate downstream signaling pathways, including PI3K/AKT, RAS/ERK/MAPK, Wnt/β-catenin, SRC, and STAT3.^20^, ^21^, ^22^, ^23^, ^24^, ^25^, ^26^, ^27^, ^28^ This can ultimately engender cellular over-proliferation, stimulate tumorigenesis, invasion, and migration, and bolster drug resistance in tumor cells.^80^

#### Abnormal *MYCN*-enhancer splicing mediated by eccDNA

Aberrant eccDNA-mediated *MYCN*-enhancer splicing can lead to *MYCN* overexpression, which exists in two distinct forms: the first form pertains to eccDNA-mediated co-amplification with noradrenergic core regulatory circuit-propelled proximal enhancers, whereas the second form of *MYCN* amplification lacks essential *in situ* enhancers and instead exhibits eccDNA usurping distal chromosomal segments carrying core regulatory circuit-driven enhancers.^81^ Furthermore, exploring pharmacological interventions aimed at eliminating eccDNA harboring *MYCN* could potentially enhance therapeutic sensitivity.^82^ Focusing on the role of *MYCN* in inhibiting anti-proliferative proteins, it is observed that CDKL5 and Dickkopf-1 are directly suppressed by this gene.^83^^,^^84^ In addition, *MYCN* facilitates the up-regulation of checkpoint kinase 1, leading to a heightened state of chemoresistance, especially notable in cases of neuroblastoma.^85^ Moreover, *MYCN*'s role extends to regulating other targets that appear to promote proliferation. An example is *MYBL2*, which is a downstream target of MYCN and is implicated in the development of drug resistance.^86^, ^87^, ^88^ In summary, irregular eccDNA-mediated shearing leads to the overexpression of the oncogene *MYCN* and abnormal activation of survival signaling pathways, fostering the emergence of drug resistance in tumors. The complex relationship between eccDNA and oncogenes like *MYCN* underscores the need for focused research to develop more effective strategies to combat tumor drug resistance.

#### eccDNA contributes to the silencing of tumor suppressor genes

Tumor cells often silence tumor suppressor genes (TSG), leading to a resistance to programmed cell death, or apoptosis, an essential step toward developing resistance to cancer treatments. It has been found that eccDNA can reintegrate itself near the *DCLK1* tumor suppressor gene, leading to its inactivation and subsequent resistance to apoptosis. Further complicating the picture, eccDNA plays a role in amplifying *MYC* genes in cases of leukemia. This amplification process suppresses the expression of miRNAs, which typically function as tumor suppressors, enhancing the ability of cancer cells to resist apoptosis. On another note, eccDNA might promote the anti-apoptotic capability of tumor cells and contribute to their drug resistance by silencing target genes through the transcription of miRNAs within the cell. This diverse role of eccDNA in cancer cells, from gene silencing to miRNA transcription, highlights its significant impact on tumor development and treatment resistance. Understanding these multifaceted roles could pave the way for more targeted and effective cancer treatments in the future.

#### The silencing of TSG *DCLK1* induced by eccDNA reintegration

**Figure 4:**
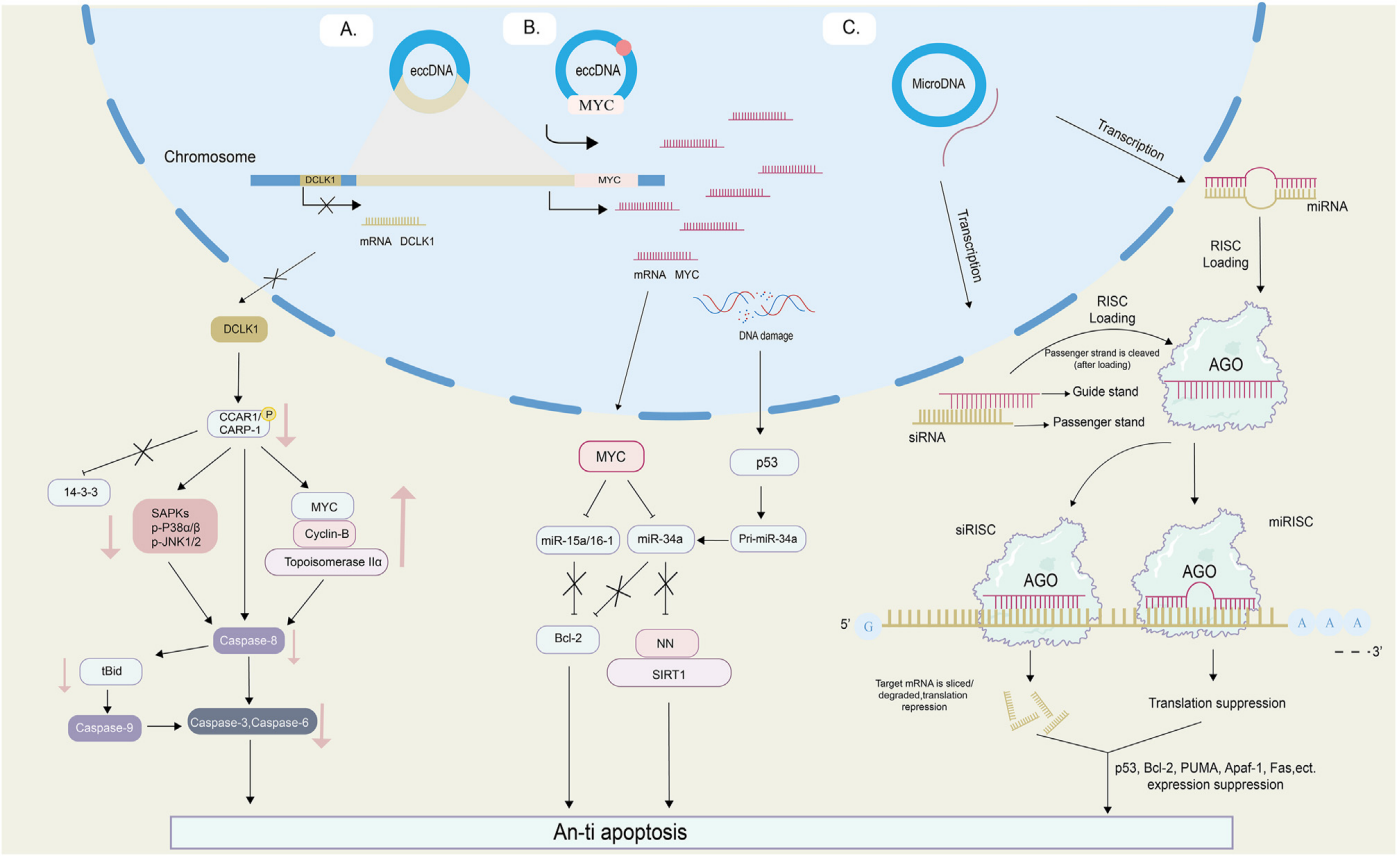
eccDNA induces tumor suppressor gene silencing, generates apoptosis resistance, and mediates cancer progression. **(A)** eccDNA-mediated suppression of oncogene DCLK1 expression leads to down-regulation of its downstream phosphorylated CARP-1/CCAR1, 14-3-3 protein, stress-activated protein kinase (SAPK), cystatin 9, and other related proteins, thereby inhibiting apoptotic signaling. **(B)** eccDNA-induced up-regulation of MYC broadly represses miRNAs (miR-34a, miR-15a, and miR-16-1), derepressing anti-apoptotic genes such as BCL2 and reasserting their anti-apoptotic effects. **(C)** Functional miRNAs directly transcribed by eccDNA silence multiple target genes (p53, Bcl-2, PUMA, Apaf- 1, Fas), producing an anti-apoptotic effect.

eccDNA can be reintegrated into the genome, and the integration sites of eccDNA and areas contained within chimeric loops are significantly enriched for genes associated with cancer (for example, the TSG DCLIK1), which displays differing expression levels when involved in the rearrangement as opposed to when it is not (Fig. 4A), and patients exhibiting such eccDNA-derived alterations tend to show poorer clinical outcomes compared with those without such modifications.^3^ This process of integrating eccDNA fragments into *DCLK1* results in loss of heterozygosity, and a prominent suppression of *DCLK1* expression. DCLK1, or dual corticotropin-like kinase 1, is a microtubule-associated protein and a member of the calmodulin-dependent kinase family.^89^ DCLK1 interacts with the cell cycle and apoptosis regulator 1 (CARP-1/CCAR1) through its C-terminal structural domain, and phosphorylates CARP-1/CCAR1 at the Ser343 site, crucial for maintaining the stability of CARP-1/CCAR1.^89^ CARP-1/CCAR1 plays roles in various biological functions such as binding to 14-3-3 proteins, stimulating the cell cycle protein-dependent kinase inhibitor p21^WAF1/CIP1^, and down-regulating cell growth and cell cycle regulators c-Myc, topoisomerase IIα, cell cycle protein B, and Rac1/p21.^90^ Therefore, in this context, rearrangements resulting from eccDNA rings can potentially lead to inactivation of tumor suppressor gene *DCLK1* expression, which in turn, enhances the anti-apoptotic capabilities of tumor cells and fosters the emergence of drug resistance. This series of complex interactions underscores the versatile roles eccDNA can play in tumor cell dynamics and provides valuable insights into potential therapeutic targets for overcoming drug resistance.

#### Regulation of TSG silencing by eccDNA through the *MYC*-miRNA circuitry

eccDNA houses a comprehensive set of genes and regulatory areas, with notable instances including oncogenes such as *MYC*, whose induced activation of *MYC* interacts intricately with miRNAs, leading to silencing of tumor suppressor genes, enhanced resistance to apoptosis, and promotion of drug resistance in tumor cells (Fig. 4B). eccDNA, as the primary form of early *MYC* amplification, could reintegrate into the chromosomal genome, inducing irregular chromosomal rearrangements and subsequently leading to the activation of *MYC* gene amplification.^91^ Further supporting this finding, eccDNA containing amplified forms of *MYC* has been observed in a variety of tumor types, including acute myeloid leukemia, granuloma carcinoma, colon cancer, and ovarian cancer.^4^^,^^92^, ^93^, ^94^, ^95^, ^96^ Additionally, both *in vitro* and preclinical evidence suggest that a reduction in *MYC* levels occurs when eccDNA is lost, resulting in enhanced therapeutic sensitivity during drug administration.^40^^,^^97^ However, *MYC* activation has been noted to broadly suppress miRNA expression, some with tumor suppressor effects, such as miR-34a, miR-15a, and miR-16-1, which is advantageous for tumor development.^98^ Moreover, in a mouse model of B-cell lymphoma, certain miRNAs suppressed by *MYC* displayed substantial anti-tumor activity.^99^, ^100^, ^101^ In conclusion, eccDNA-induced activation of *MYC* leading to the silencing of oncogenes can bolster resistance to apoptosis in cancer cells, fostering the emergence of drug resistance in these cells. This intricate interaction between eccDNA, *MYC*, and miRNAs provides a comprehensive view of the complex gene regulatory networks in cancer cells and underscores potential targets for more effective therapeutic interventions.

#### Regulation of TSG silencing by eccDNA at the miRNA level

eccDNA carries out gene expression regulation by generating functional miRNA and siRNA through transcription within the cell (Fig. 4C). MicroDNA (a type of eccDNA) carries transcripts of miRNA genes, which are independent of traditional promoter sequences, and can be transcribed *in vitro* and *in vivo* to the expression of functional small regulatory RNAs such as microRNA and novel siRNA, while newly formed siRNAs can repress endogenous genes in microDNAs, which is related to the RNA polymerase subunits POLR2H and POLR3F.^8^ Given the function of microDNA carrying miRNA genes, it is likely that they play a pivotal role in regulating biological processes. Many dysregulated miRNAs enhance the ability of tumors to resist cell death, when *p53*-mediated transcriptional repression is inhibited under hypoxic conditions, tumor cells expressing elevated levels of miR-17-92 might evade hypoxia-induced apoptosis.^102^ The miR-221/222 molecules inhibit apoptosis in human gliomas by targeting *PUMA*, an apoptosis promoter.^103^ Moreover, miR-21 is up-regulated in cancers and inhibits the apoptosis pathway by controlling extrinsic components of the pathway (by inhibiting *Apaf-1* expression and reducing Fas protein levels).^104^ Therefore, eccDNA might contribute to drug resistance in tumor cells by silencing target genes through the generation of functional miRNA pathways with effects such as anti-apoptosis. This complex interplay between eccDNA, miRNA, and gene silencing, provides a more comprehensive understanding of drug resistance in cancer, with potential implications for future therapeutic strategies.

#### Post-transcriptional regulation of eccDNA by affecting protein–protein interactions

**Figure 5:**
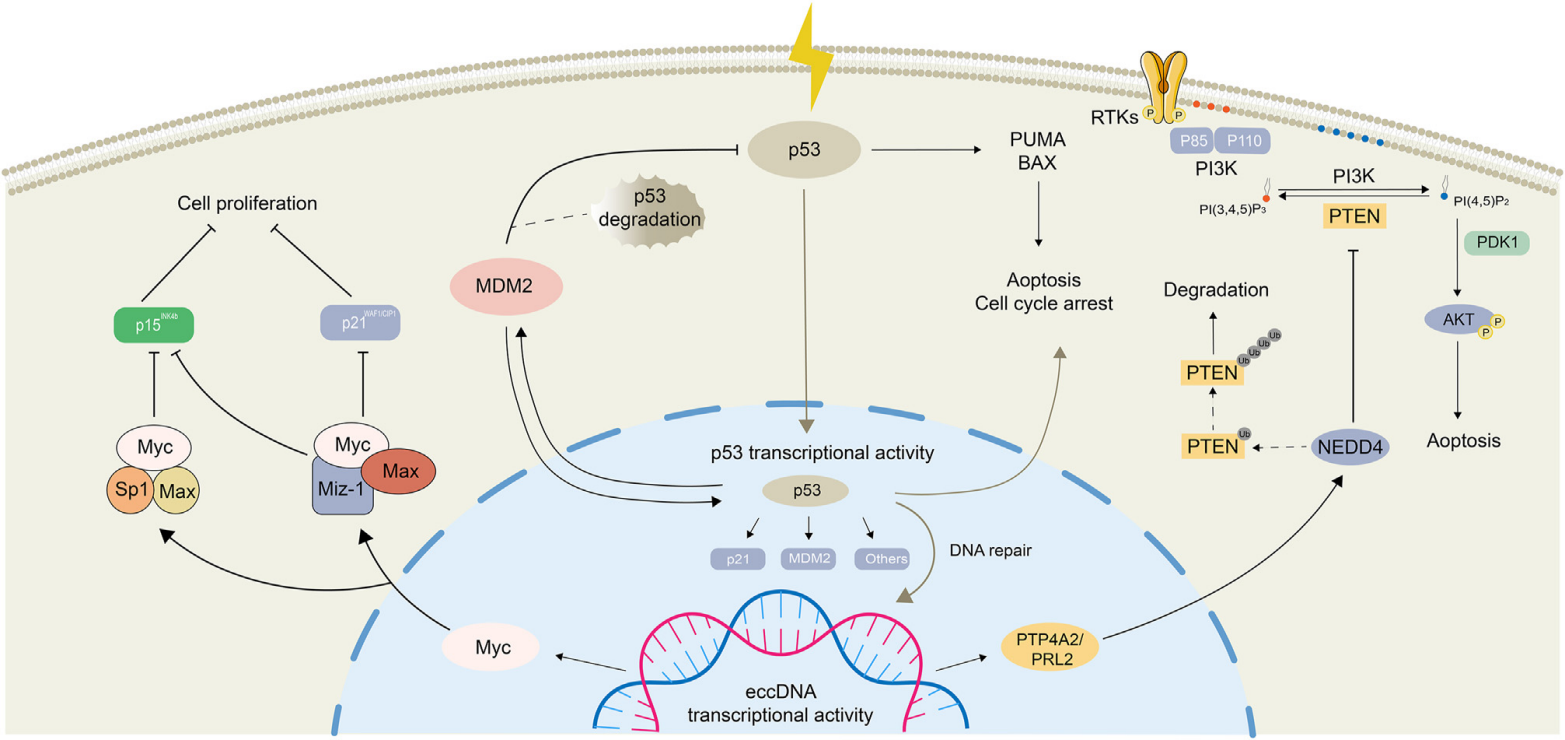
eccDNA affects protein interactions and mediates cancer progression. eccDNA-mediated MYC expression in tumor cells inhibits the activity of zinc finger protein 1 (Miz1) through protein interaction, which further prevents the activation of tumor oncogenes by Miz1 and produces an anti-apoptotic effect, leading to cancer progression. Myc can also inhibit activities such as Sp1 through protein interactions, accelerating tumor progression and leading to therapeutic resistance in tumor cells. In addition, eccDNA-induced MDM2 overexpression inhibits p53, which blocks p53 through three mechanisms: i) it directly inhibits p53-mediated transcriptional activity by binding to its transcriptional activation domain; ii) hence, MDM2 prevents p53 from binding to target DNA by binding to the nuclear export signal sequence of p53; iii) MDM2 promotes ubiquitinated degradation of p53 by acting as an E3 ubiquitin ligase,^114^ which in turn leads to anti-apoptotic effects in tumor cells and promotes cancer progression. Conversely, eccDNA-mediated PTTA2A expression in tumor cells promotes AKT signaling in tumor cells by mediating the degradation of PTEN ubiquitination, which increases tumor cell survival and leads to cancer progression.

eccDNA is also regulated post-transcriptionally at the protein level in tumor cells. eccDNA can be involved in a variety of protein–protein interactions that lead to loss of dimerization and subsequent degradation of cancer-associated proteins, thereby causing a "butterfly effect" that promotes drug resistance, which affects the therapeutic efficacy of tumor therapy. Notable examples of this phenomenon include Myc and Miz1, MDM2 and p53, and PTP4A2-PTEN (Fig. 5).

#### The affection of eccDNA on Myc and Miz1 protein–protein interactions

As previously stated, the transcription factor Myc, the transcription product of the oncogene *MYC* induced by eccDNA activation, can be drawn to the core promoter via protein interactions, thereby mitigating positive transcriptional regulation.^105^ In the specific case of the Mic-interacting zinc finger protein 1 (Miz1), Myc has the ability to bind to it and inhibit its activity. This interaction subsequently renders Miz1 unable to activate proteins that otherwise serve to repress cell cycle initiation, such as CDKN1A (p21^WAF1/CIP1^) and CDKN2B (p15^INK4b^).^106^^,^^107^ In addition to Miz1, Myc can interact with and counteract the effects of other transcription factors, including Sp1. Such interactions can spur the progression of tumors and foster resistance to drugs within tumor cells.^108^, ^109^, ^110^, ^111^ Therefore, the interaction between Myc and other transcription factors such as Miz1 and Sp1 is a key factor in promoting tumor growth and drug resistance. Understanding these interactions could prove vital in developing new strategies to combat cancer, by identifying potential therapeutic targets that could disrupt these relationships and reverse the resistance caused by Myc's influence.

#### The affection of eccDNA on MDM2 and p53 protein–protein interactions

eccDNA-mediated *MDM2* amplification acts as a compensatory mechanism to promote drug resistance in anti-EGFR therapy. Resistance to anti-EGFR treatment might not be solely attributed to *EGFRvIII* positivity; resistant cells carry large amounts of MDM2-positive eccDNA and MDM2^+^ eccDNA expression continues to surge after administration of erlotinib and remains high for a period after the drug's discontinuation.^21^ *MDM2* is considered a primary mechanism to inhibit wild-type *p53* as part of a self-regulatory loop, wherein each modulates the other's cellular levels; in more detail, MDM2 subverts p53-mediated apoptosis by marking p53 for processes like ubiquitin-mediated degradation; this shift in the balance of pro- and anti-apoptotic proteins allows cancer cells, which would normally undergo apoptosis, to survive and build resistance to treatment.^112^ In this context, eccDNA, through the mediation of *MDM2* amplification, can influence the interaction between MDM2 and p53, affecting p53 degradation, and subsequently bolstering tumor drug resistance. This detailed exploration of the role of eccDNA in mediating *MDM2* amplification and its influence on tumor drug resistance underlines the complex relationship between eccDNA and key proteins in the p53 pathway, providing fresh insights for developing therapeutic strategies against cancer.

#### The affection of eccDNA on PTP4A2 and PTEN protein–protein interactions

eccDNA has been associated with the amplification of *PTPA2* in neuroblastoma; it mediates chimeric loop formation where the oncogene *PTP4A2* is cyclized and co-amplified with adjacent enhancers, and the full integration of *PTP4A2* on cyclized DNA and amplification as eccDNA resulted in a substantial increase in its expression.^3^ PTP4A2/PRL2 is a regenerating liver phosphatase, and PTP4A2 can down-regulate PTEN through Y336 dephosphorylation in PTEN, causing PTEN to be ubiquitinated and degraded by NEDD4.^113^ Thus, the eccDNA-mediated up-regulation of PTP4A2 decreases PTEN levels and increases AKT signaling, leading to decreased survival and ultimately promoting drug resistance in tumor cells.

## Conclusion and prospect

eccDNA is prevalent in a vast majority of human cancer cells. Its enrichment typically facilitates the amplification of oncogenes, thereby enhancing their plasticity and instability which is integral to the evolution of tumor cells. Existing research underscores the presence of both oncogenes and drug-resistance genes in tumor extrachromosomal DNA. However, the specific processes leading to drug resistance require further elucidation. This review aims to delve into eccDNA's role in promoting tumor growth and its contribution to tumor therapeutic effect (Table 1). The unequal partitioning of eccDNA intensifies the heterogeneity among tumors. In addition, eccDNA enhances the “stemness” of tumor cells by regulating genes associated with tumor stem cells as well as signaling pathways, thereby contributing to the maintenance of malignancy and accelerating tumor cell progression. Another crucial aspect is eccDNA's role in modulating the tumor immune microenvironment by controlling the release of cellular signaling molecules, thereby fostering immune tolerance in tumor cells. eccDNA possesses an inherent immunostimulatory effect which induces the production of several inflammatory factors that serve to regulate the microenvironment. The powerful role of eccDNA in tumor progression and therapy may be attributed to its complex regulatory mechanisms on tumor cells. Through chimeric cyclization and reintegration into the genome, eccDNA promotes the aberrant production and expression of oncogene shedders, leads to the remodeling of the cancer genome, and triggers the abnormal activation of survival signaling pathways. EccDNA's chimeric cyclization further results in the silencing of oncogenes, which produces anti-apoptotic effects. Moreover, eccDNA mediates interactions among specific proteins, influencing their degradation. Despite significant progress, several aspects of eccDNA research harbor unresolved questions and unexplored territories that warrant further discussion.

**Table 1:** Mechanisms regulating cancer progression by eccDNA.

Study level	Mechanism	Factor	Key findings	References
Function study	eccDNA-mediated tumor heterogeneity	Lack of centromere and unequal division	eccDNA's unequal division always reversibly drives up-regulation or inhibition of mutant epidermal growth factor receptor (EGFR), emerges as tumor heterogeneity, and promotes tumor therapeutic resistance.	2,10,21
	eccDNA-mediated mechanisms driving tumor cell stemness and therapeutic resistance	EGFR	eccDNA amplifies EGFR, promoting glioma stem cell (GSC) self-renewal and enhancing tumor cell stemness, ultimately advancing therapeutic resistance.	2,38
		c-Myc	eccDNA amplifies c-myc, playing a role in maintaining GSCs and tumor cell stemness, contributing to therapeutic resistance.	39, 40, 41, 42
		Autophagy	eccDNA-encoded RAB3B induces autophagy, potentially intensifying tumor cell stemness and enhancing resistance to cancer treatments.	43, 44, 45, 46
	eccDNA-mediated regulation of the tumor immune microenvironment and its impact on immunotherapy efficacy	Type I interferons	eccDNA activates the STING-dependent pathway, leading to the production of type I interferons, enhancing cytotoxic T cell activity and immunotherapy efficacy.	52, 53, 54, 55, 56, 57, 58, 59, 60
		Interleukin (IL)-6 and tumor necrosis factor-alpha (TNF-α)	eccDNA-induced IL-6 and TNF-α production contributes to an immunosuppressive microenvironment, reducing immunotherapy effectiveness.	61, 62, 63, 64, 65, 66, 67, 68
		SOX11	eccDNA-mediated SOX11 overexpression hinders B-cell maturation, impacting immune cell composition in the tumor immune microenvironment and diminishing immunotherapy efficacy.	3,69, 70, 71
		Neurotrophin-3	eccDNA amplifies neurotrophic factor-3, inhibiting microglia activation and promoting tumor cell survival at metastatic sites, reducing immunotherapy efficacy.	3,72,73
Mechanistic study	eccDNA-mediated abnormal splicing and oncogene amplification in cancer	EGFR-enhancer	eccDNA-driven localized abnormal splicing in glioblastoma leads to EGFR amplification. This abnormality activates EGFR, promoting tumor cell survival, proliferation, and therapeutic resistance.	6
		MET-Capza2	eccDNA-mediated MET-Capza2 fusion in glioblastoma results in MET oncogene overexpression. Variability in splice patterns enhances downstream pathways, driving tumor proliferation, invasion, and therapeutic resistance.	5
		MYCN-enhancer	eccDNA-induced MYCN overexpression involves the hijacking of enhancers. Two distinct forms of eccDNA-mediated MYCN amplification contribute to therapeutic resistance by affecting anti-proliferative proteins, checkpoint kinases, and downstream targets that promote tumor growth.	81,82
	eccDNA-mediated regulation of tumor suppressor genes and miRNA pathways in cancer	Dual corticotropin-like kinase 1 (DCLK1)	eccDNA integration into tumor suppressor gene DCLK1 results in its inactivation, enhancing the anti-apoptotic capabilities of tumor cells and promoting therapeutic resistance.	3
		MYC	eccDNA amplification of MYC leads to the silencing of miRNAs, typically functioning as tumor suppressors, thus bolstering cancer cell resistance to apoptosis and therapeutic resistance.	4,40,91, 92, 93, 94, 95, 96, 97
		miRNA	eccDNA generates functional miRNAs and siRNAs, contributing to gene silencing. Dysregulated miRNAs in cancer cells enhance anti-apoptotic pathways, aiding tumor resistance to cell death and therapy.	8
	eccDNA-mediated protein interactions and their impact on tumor growth	Myc and Miz1	eccDNA-mediated interactions with Myc and transcription factors like Miz1 promote tumor growth and therapeutic resistance.	105
		MDM2 and p53	eccDNA-driven MDM2 amplification affects the MDM2-p53 interaction, contributing to therapeutic resistance in cancer cells.	21,112
		PTP4A2 and PTEN	eccDNA-mediated PTP4A2 amplification reduces PTEN levels, increasing AKT signaling and promoting therapeutic resistance.	3,113

To begin with, the detailed mechanisms and direct evidence surrounding the biological formation of eccDNA are areas requiring additional scrutiny. The exact catalyst that leads to eccDNA formation remains a mystery. Previous research indicates that eccDNA can originate from processes that fragment chromosomes, yet the methodology through which these linear chromosome fragments undergo cyclization is still undetermined. Additionally, we are yet to establish uniform classification criteria for eccDNA, and understanding its spatial topology remains a challenge. Also, it is not fully understood if determining the structure of eccDNA impacts its stability. This highlights the fact that while we have gleaned some understanding of eccDNA, there are still significant gaps in our knowledge that necessitate further exploration.

Moreover, the regulation and precise functionality of eccDNA in the context of cancer development still require more solid validation. Numerous studies have zeroed in on the gene amplification potential of large eccDNA. Also, while eccDNA's contribution to the reorganization of the cancer genome through cyclization and reintegration has been recognized, the characteristics of many types of eccDNA remain largely unexplored. In the past, loop-derived rearrangements were somewhat overlooked in genomic sequencing analyses. It has been proposed that a proportion of eccDNA exists within the nucleus and can undergo transcription. However, further verification is needed to confirm whether the microRNAs produced from such transcriptions partake in gene regulation, as suggested by Paulsen and colleagues in 2019, and whether these have a role in promoting tumorigenesis. This reinforces the necessity for deeper investigations into the multi-faceted roles of eccDNA.

While researchers acknowledge the substantial impact of eccDNA on cancer initiation and progression, the exploration, application, and evolution of this knowledge remain in the nascent stages. A wealth of questions within this field remains unanswered. Furthermore, non-toxic methodologies aimed at eradicating eccDNA have shown promise in addressing the issue of treatment resistance among cancer patients. However, the dynamic mechanics behind these strategies require further clarification. There should be an increased emphasis on understanding how eccDNA affects cancer progression and subsequent treatment and the underlying mechanisms. This could potentially pave the way for the development of innovative therapeutic approaches to improve cancer treatment efficacy.

## Author contributions

Siqi Zheng (First Author): Conceptualization, Methodology, Software, Investigation, Formal Analysis, Writing–Original Draft; Yunong Li: Data Curation, Writing–Original Draft; Lin Wang: Visualization, Investigation; Qian Wei: Resources, Supervision; Minjie Wei: Software, Validation; Tao Yu^∗^: Visualization, Writing–Review & Editing; Lin Zhao∗: Conceptualization, Funding Acquisition, Resources, Supervision, Writing–Review & Editing.

## Conflict of interests

The authors declare that they have no conflict of interests.

## Funding

This study was supported by grants from the 10.13039/501100001809National Natural Science Foundation of China (No. 82073281, 81573462, 82073884, U20A20413), Shenyang S&T Projects (Liaoning, China) (No. 20–204–4–22), Science and Technology Innovation Team Project of China Medical University (No. CXTD2022007), and Supporting the High-Quality Development of Science and Technology Funding Projects at 10.13039/501100007300China Medical University (No. 2022011963-JH2/202).
